# ERP and attachment dimensions as predictors of seeking care or food comfort in stressful situations

**DOI:** 10.1038/s41598-023-29493-0

**Published:** 2023-02-23

**Authors:** Arcangelo Uccula, Beniamina Mercante, Caterina Pozzati, Franca Deriu, Paolo Enrico

**Affiliations:** 1 Department of History, Human Sciences and Education, University of Sassari, Via Zanfarino 62, 07100 Sassari, Italy; 2 Department of Biomedical Sciences, University of Sassari, Viale San Pietro 43/B, 07100 Sassari, Italy; 3 Unit of Endocrinology, Nutritional and Metabolic Disorders, AOU Sassari, Sassari, Italy

**Keywords:** Neuroscience, Physiology, Psychology

## Abstract

In this study, we aimed to test the association of the Late Positive Potential (LPP) response and attachment dimensions in the choice of care/food pictures and its reaction time (RT) in threatening versus neutral conditions. Fifty-two participants (38 females, M_age_ 22.62) responded to the ECR questionnaire and were exposed to adequate visual stimuli, during EEG recording. Results showed that threatening stimuli increase the choice of care, decrease RT, and increase LPP magnitude in centro-parietal areas (Cpz, Pz, P3 and P4). Food choice was lower, with increased RT. Furthermore, larger LPP magnitude in centro-parietal cluster was associated with increased RT in the choice of care. Considering the dimensions of attachment, in threatening conditions, while anxiety was not associated with RT and care/food choice, avoidance was associated with an increase in care choice and RT. In conclusion, the specific association of increased RT in care choice with high LPP magnitude centro-parietal cluster may be explained in terms of a functional interference of these areas in the choice of care, but not of food. Further, we postulate that the increased RT of avoidant individuals may reflect a more articulated choice process.

## Introduction

Attachment theory explains the children's construction of a preferred relationship with a protective figure to increase survival when in danger and in times of need. The core of the attachment theory is the alleviation of distress and fear from a perceived environmental threat, through a process of co-regulation provided by a caregiver^1^. More specifically, when a threat activates the attachment system, it automatically motivates the subject to seek physical or symbolic proximity to an attachment figure, which can provide a sense of safety, helping to cope successfully with threats^2^. According to Bowlby^1^, interactions with attachment figures are remembered in the form of a schematic internal working model^3^, and are automatically carried out when processing new information^4^. These representations consist of both conscious and unconscious memories of interaction with attachment figures, as well as strategies for regulating negative emotions^5^. In adulthood, the primary attachment strategy is not necessarily the behavior of seeking physical proximity, but it may also consist in the activation of mental representations of their symbolic presence^6^.

Individual differences in the level of attachment system activation have generally been conceptualized in terms of two dimensions: anxiety and avoidance, which are associated with distinct patterns of emotional distress regulation. When confronted with salient stimuli, individuals with anxious attachment show patterns of hyperactivation, while avoidant attached individuals show patterns of deactivation^6,7^. An event-related potentials (ERPs) study^8^ found that avoidant attached adults exhibited a bias toward increased neural activation, showing greater motivational relevance and attention to negative emotional stimuli. This result, which appears in contrast to the deactivation assumption of avoidant individuals, may be explained through a dual process in which avoidance behavior follows awareness of the nature of the stimulus^9^, necessary in order to activate the avoidant defense.

ERP research has amply demonstrated that the late positive potential (LPP) response is enhanced for salient stimuli and reflects relatively deep encoding and cognitive processing^10–12^. Bradley^13^ argues that the LPP may represent a neural response indicating that relevance has been detected in the environment and, therefore, emotional content elicits an increase in LPP because it activates aversive or appetitive motivational systems. These findings are consistent with the cognitive models that emphasize the role of schema-driven processing in allocating attention to threatening stimuli^14^.

The processing of emotional information and its regulation has been investigated by several researchers^15,16^. A convergence emerges from the studies regarding the role of attentional and memory processes in terms of accessibility to mental concepts. According to priming research, primes often do not cause direct effects, but rather affect the accessibility of mental content related to the primes. The impact of affective priming should depend on the nature of the accessible content and its relation to the current task. Therefore, affective priming could modulate decisions through several pathways, including non-decision time and bias^17^. Mikulincer et al.^18^ found that threatening primes led to greater accessibility of mental representations of the attachment figure, as indicated by faster reaction times (RT). Later, Dewitte et al.^19^ demonstrated that priming with attachment-related threats, automatically activated an attention bias toward the attachment name figure. Conversely, studies of attention to positive stimuli indicate that the magnitude of attentional bias varies in proportion to motivational variables such as relevance and arousal^20,21^. The magnitude of attentional bias is also consistent with the incentive salience hypothesis, in which positive stimuli capture attentional resources^22^. In this perspective, various authors used positive stimuli with a higher level of biological relevance such as nutrition, offspring care, and reproduction^23^. Relevant negative and positive stimuli are then attention trigger signals and fulfill different and complementary motivational goals. Aversive stimuli generate activation and drive to action, while approach stimuli can play a regulatory function and restore homeostasis^16,24^.

Overall, extensive research described the effect of threatening stimuli on the physiological response as indexed by LPP amplitude as well as on the propensity to seek care. However, there is less experimental evidence on the association between attachment dimensions, LPP amplitude and emotion regulation through comfort seeking, in threatening situations.

Therefore, in this work we studied whether care-seeking is the primary choice after exposure to threatening stimuli, and whether the participants' different attachment orientations lead their choice toward other forms of emotion regulation, such as comfort food (henceforth: food)^25^. Parental child care pictures (a strong symbolic representation of care), in agreement with attachment theory, were used as the goal-congruent stimulus to the threatening condition. Food, on the other hand, was selected as the alternative choice because of its strong biological reward function, irrespective of the eating style^26^, for its enhanced attentional effect^27^ and for its role in the regulation of negative emotional states and restoration of homeostasis^28–30^ related to the attachment system^31^.

Based on the attachment theory and studies on attentional bias, we expect that in a threatening situation participants will generally choose a stimulus congruent with the negative emotional trigger, such as a care picture. On this line, we also hypothesize that the choice of care, but not the choice of food, will be associated with a shorter RT in comparison to the neutral condition. Further, despite food being an attentional priority and its emotion regulation function, in our experimental setting it should be a secondary choice and therefore associated with a longer RT in comparison to the neutral condition.

Based on previous research we also focused on two aspects potentially relevant to the process of choice and its RT. The first concerns attachment representations in the two dimensions of anxiety and avoidance, the second concerns LPP magnitude. We expect that attachment anxious individuals, hypervigilant to emotional stimuli, would be more motivated to seek care, with a shorter RT in comparison to individuals with low anxiety. Regarding avoidant individuals, recent evidences have shown that, despite they rely on attachment deactivation as a defense when coping with threats, when they are certain of the availability of another person, they actually activate the proximity-seeking behavior^32^. However, due to their threatening information processing^9^, it is hypothesized that avoidant individuals will show an increase in RT, specifically when choosing care.

Finally, being the LPP an indicator of emotional response, we expect to record an increased magnitude in the threatening condition, and hypothesize that this phenomenon will be associated with an increase in care choice. We also hypothesize that differences in LPP magnitude between the neutral and threatening conditions will be associated with an increase in RT, because of the interference of the high emotional activation on the decision making process.

## Methods

### Participants

Fifty-two healthy subjects (14 males and 38 females; mean age 22.62 ± 2.82 years (range = 19–28 years) participated in the study. A written informed consent was obtained from all subjects prior to study entry; the experimental procedure was approved by the Institutional Review Board of the Department of Biomedical Sciences of the University of Sassari and conducted in accordance with the Helsinki Declaration. None of the participants had history and/or current signs/symptoms of neurological and/or psychiatric diseases and current or recent use of any drugs affecting the central nervous system. All participants had normal or corrected-to-normal vision.

### Experimental procedures

The study was composed of two experimental sessions: questionnaires administration and EEG recording.

#### Questionnaire

Attachment style was measured using the Italian version^33^ of the Experiences in Close Relationships Scale revised (ECR-R^34^). This questionnaire contains 36 relationship-related statements that refer to attachment anxiety and avoidance. The anxiety scale includes eighteen items that reflects an individual’s concern about rejection and abandonment. The avoidance scale includes eighteen items that assesses discomfort with closeness and dependence. Participants responded on a 7-point Likert scale ranging from 1 (strongly disagree) to 7 (strongly agree).

In the current sample, Cronbach alphas were high for the anxiety scale, *α* = 0.92, as well as for the avoidance scale, *α* = 0.91. The mean attachment scores were 3.16 (*SD* = 1.17) for attachment anxiety and 2.33 (*SD* = 0.93) for attachment avoidance.

Participants filled in the ECR-R questionnaire and underwent EEG recording in a counterbalanced way: half started with the questionnaire and half started with the recording.

#### EEG recordings

EEG recordings were carried out in a quiet, dimly lighted room, while subjects were seated in a comfortable chair and were asked to stay relaxed but alert during the experiment. EEG data was collected using a Neuroscan Synamps amplifier (Compumedics, Charlotte, CN, USA), digitized (16-bit resolution, 1 kHz sampling frequency), band-pass filtered (0.05–100 Hz), and recorded using Scan software (v 4.3, Compumedics, Charlotte, CN, USA). EEG recordings were obtained with standard 32-channels Ag/AgCl electrodes arranged according to the 10–20 international EEG system, and included Fp1, Fp2, F7, F3, Fz, F4, F8, Ft7, Fc3, Fcz, Fc4, Ft8, T3, C3, Cz, C4, T4, Tp7, Cp3, CPz, CP4, Tp8, T5, P3, Pz, P8, T6, O1, Oz, O2. Averaged mastoids (2 electrodes placed over the mastoid bones) served as the reference lead and the ground electrode was placed on FPz. For electrooculogram recordings, electrodes were placed above and below the left eye, as well as on the outer canthi of each eye. Skin impedances were kept below 5 kΩ, and during the experiment the EEG signal was continuously monitored online.

Off-line EEG processing was performed with EEGLAB R2019b update 7^35^ and ERPLAB^36^ toolboxes running in the MATLAB environment (Version 2019b, MathWorks, Inc., Natick, MA, USA). Raw EEG signal was epoched (− 300 to 1200 ms, timelocked to stimulus onset) and demeaned (baseline: − 300 to 0 ms). Epochs were visually inspected and excessively noisy ones excluded from analysis (less than 5% for each participant). Residual artifacts were identified using an Independent Component Analysis algorithm (INFOMAX ICA) and eliminated after visual inspection, based on time, frequency, scalp distribution, and amplitude criteria.

For the purposes of the study CPz, Pz, P3 and P4 channels (centro-parietal cluster) were selected for analysis^37^. LPP peak was identified as a positive deflection with latency between 500 and 1000 ms and its magnitude measured by calculating the relative area under curve.

#### Visual stimuli administration

Visual stimuli were presented using STIM2 software (Compumedics, Charlotte, CN, USA), on an analog 17’ color monitor at a viewing distance of approximately 60 cm. Pictures occupied about 40° of visual angle horizontally and vertically, and participants were instructed to fixate on the center of the screen. In order to record the picture chosen as well as the RT, subjects were instructed to use a dedicated response pad (Compumedics, Charlotte, CN, USA) to choose between the target pictures. RT was defined as the time elapsed between the stimulus presentation and the key press.

Two hundred and seventy pictures were selected from the following databases to serve as threatening stimuli or target pictures in the experiment:

- For emotional stimulation, the International Affective Picture System (IAPS) ((IAPS): Threatening: 1120, 1304, 3010, 3015, 3030, 3061, 3063, 3064, 3071, 3100, 3102, 3110, 3120, 3130, 3140, 3225, 3230, 3500, 3530, 6313, 6315, 6350, 6370, 6510, 6550, 6560, 6570, 9253, 9414, 9635. Neutral: 7000, 7002, 7004, 7009, 7011, 7012, 7018, 7019, 7020, 7025, 7026, 7032, 7040, 7042, 7050, 7053, 7059, 7061, 7062, 7080, 7090, 7095, 7150, 7175, 7185, 7190, 7205, 7211, 7233, 7235.) was used^38^. A total of 60 IAPS pictures were selected for the threatening (e.g. mutilation, accidents, human attack, illness) and neutral (e.g. domestic objects) conditions. The contents were associated consistently with higher or lower valence or arousal ratings. Mean ratings of valence and arousal were as per Lang et al., 2008 (9 points Likert-like scale: 1 = low, negative). The 60 pictures had a mean valence of 2.05 (*SD* = 0.54) and *M* = 5.07, (*SD* = 0.32); mean arousal of 6.52 (*SD* = 0.50) and *M* = 3.0 (*SD* = 0.64) for threatening and neutral condition respectively. Valence and arousal were significantly different between the two conditions, *F*(1, 38) = 264 and *F*(1, 38) = 438 respectively.
- For one of the two choice options, 60 pictures from the The Besançon Affective Picture Set-Adult (BAPS-Adult^39^) ((BAPS). Care (threatening condition): 1, 5, 6, 8, 9, 12, 14, 15, 16, 17, 25, 27, 29, 30, 31, 32, 34, 36, 39, 40, 41, 43, 47, 48, 49, 52, 53, 54, 55, 61. Care (neutral condition): 2, 3, 4, 7, 11, 13, 19, 20, 21, 22, 23, 24, 26, 28, 35, 37, 38, 42, 44, 46, 50, 51, 56, 57, 58, 59, 60, 62, 63, 64.), were used. The 60 pictures depicted comfort-related scenarios where care was represented (e.g., an adult comforts an infant in distress). Two random lists of 30 pictures were created: one for the threatening and one for the neutral condition. Mean ratings of perceived care, valence and arousal, were as per Szymanska et al.^39^: the two lists did not significantly differ on any dimension, all *F* values < 1.
- For the other of the two choice options, 60 pictures were taken from the Food-Pics_Extended^40^ ((Food-Pics). Food (threatening condition): 4, 5, 9, 14, 22, 26, 45, 56, 66, 67, 90, 94, 104, 109, 116, 133, 137, 170, 177, 186, 189, 225, 287, 539, 613, 673, 684, 713, 877, 878. Food (neutral condition): 15, 25, 31, 35, 38, 43, 44, 60, 65, 80, 102, 103, 113, 115, 121, 127, 130, 138, 140, 150, 172, 173, 183, 205, 286, 510, 614, 676, 682, 869.). The pictures were of typical foods (sweet and salty snacks) and they were randomly divided into two random lists of 30 items: one for the neutral and one for the threatening condition. Mean ratings of calories, palatability, and craving were as per Blechert et al.^40^: the two lists did not significantly differ on any dimension, all *Fs* < 1.
- For the 30 filler-trials 90 neutral pictures were selected from the IAPS and Food-Pics_Extended databases.

**Figure 1:**
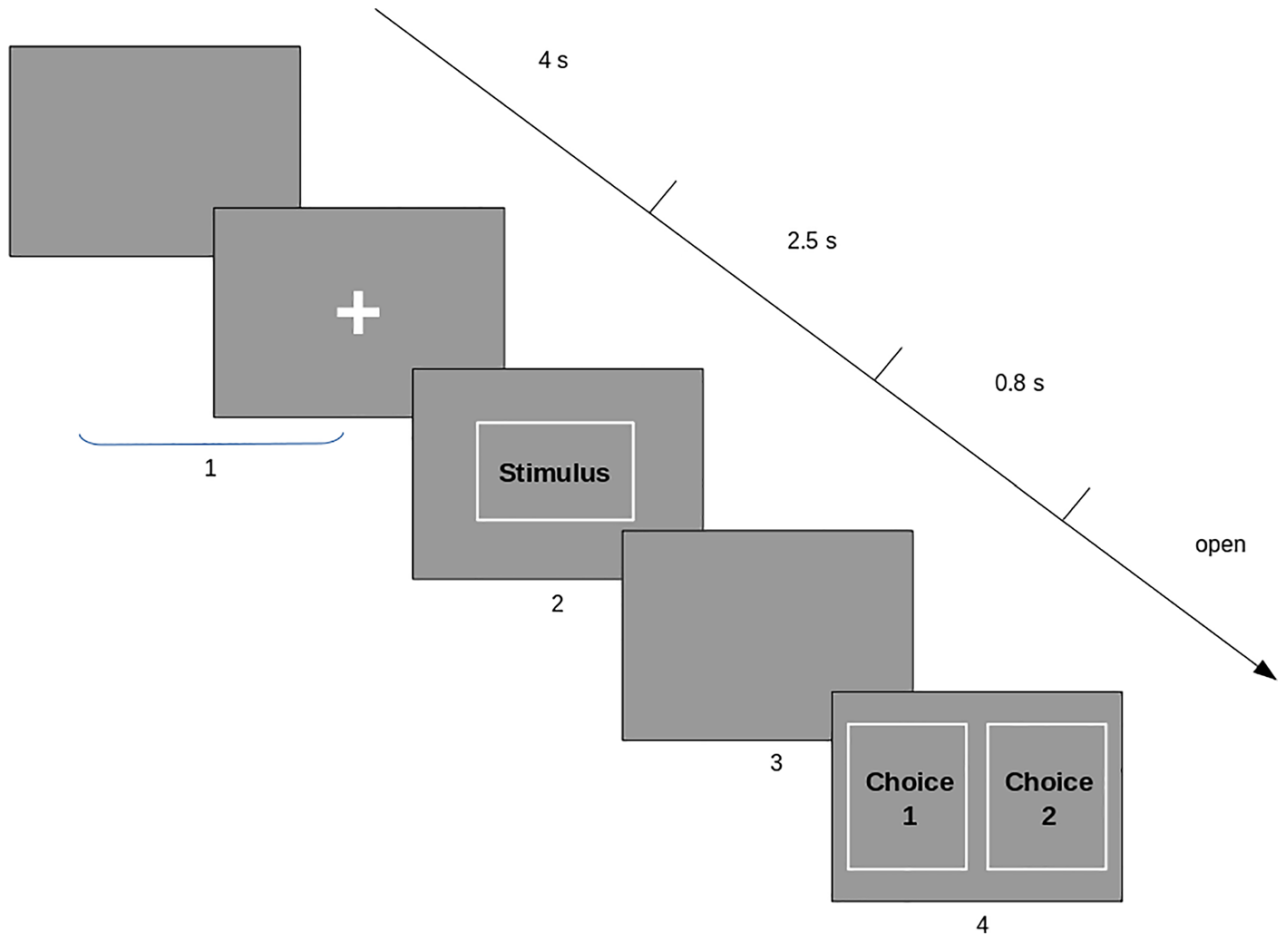
Experimental task used in the study, showing from top left to bottom right the progression of the visual stimuli in time. At the start of each trial a gray screen followed by a fixation cross is displayed (total time 5 s), then the threatening stimulus is displayed in a central location (2.5 s), followed by another gray screen (0.8 s), after which two probe pictures are displayed side-by-side.

The experiment consisted of 90 randomly intermixed trial: 30 trials in the threatening condition; 30 trials in the neutral condition; and 30 filler trials. Each trial began with a gray screen followed by a fixation dot in the center of the screen; the duration of the two pictures varied randomly up to a total duration of 5 s, followed by a prime picture (threatening or neutral) lasting for 2.5 s, another gray screen lasting for 0.8 s, after which two probe pictures were presented side-by-side (Fig. 1). Both in the neutral and in the threatening condition, one of the two pictures depicted a caring scenario whereas the other one depicted food. In the filler trials the two probe pictures had a neutral content. Probe pictures remained visible until participants responded. The relative position of the two pictures (left vs. right) was counterbalanced^41^.

Prior to the start of the task, participants were adequately informed on the experimental procedure; to ensure consistency, all information on the experimental procedure were administered as part of the visual stimulation protocol (STIM2 Compumedics, Charlotte, CN, USA). Participants were allowed to familiarize with the experimental procedure with 10 practice trials; all pictures in the practice trials had neutral content, and were not included in the experiment.

#### Statistical analyses

Repeated measures analyses of variance (ANOVA) with the factors of condition (2) × electrocortical position (4) was performed. Huynh–Feldt corrected degrees of freedom were applied when the assumption of sphericity was not met.

To compare the care/food choice and the RT differences, separate two-way repeated-measures ANOVA were performed between the neutral and threatening condition.

We also conducted four hierarchical multiple regression analyses (four-step) to assess the association between the choice of care and food and their RT as dependent variables and anxiety attachment, avoidance attachment and LPP magnitude differences between conditions as predictors. LPP magnitude in the centro-parietal cluster, care/food choice and RT scores used in the regression analyses, were determined by subtracting the averages of the neutral condition from the threatening condition.

In the first regression analysis, RT differences, attachment anxiety, attachment avoidance, and LPP differences were entered as predictors, and the care choice differences as a dependent variable. In the second regression analysis the care choice differences, attachment anxiety, attachment avoidance, and LPP differences were entered as predictors; while the RT differences as a dependent variable. In the third regression analysis, RT differences, attachment anxiety, attachment avoidance, and LPP differences were entered as predictors, the food choice differences as a dependent variable. In the fourth regression analysis the food choice differences, attachment anxiety, attachment avoidance, and LPP differences were entered as predictors; while the RT differences as a dependent variable.

The order of entry of predictors was based on evidence and theory described earlier. Since RT and care/food choice have been associated with each other, in the first regression analysis (dependent variable: care choice), RT was included first; in the second regression analysis (dependent variable: RT) care choice was included first^42^. The same analysis structure was also applied in the third and fourth regression, using food choice and RT as dependent variables. The hierarchy of inclusion of the remaining variables are common to all regression analysis. Attachment anxiety was entered at Step two because it is a central component of attachment theory under threatening situations. The avoidance dimension of attachment was included in Step three. In step four centro-parietal cluster were entered, along with the other predictors.

## Results

Experimental values across the two conditions and their differences are reported in Table 1.

**Table 1:** RT, Choice, and LPP magnitude values in the threatening and neutral conditions, and their differences. Data are expressed as mean ± SD.

Variables	Neutral (N)		Threatening (T)		Differences (T–N)	
	Care	Food	Care	Food	Care	Food
RT (ms)	2623.89 ± 1355.39	2524.86 ± 1162.42	2258.17 ± 1042.36	3072.82 ± 1806.40	− 365.72 ± 869.67	547.97 ± 1036.88
Choice	16.29 ± 7.39	13.71 ± 7.39	22.96 ± 7.23	7.04 ± 7.23	6.67 ± 7.47	− 6.67 ± 7.47
Cpz	1.69 ± 1.31		6.14 ± 2.38		4.45 ± 1.86	
Pz	1.62 ± 1.15		6.01 ± 2.02		4.39 ± 1.73	
P3	1.71 ± 1.24		5.74 ± 2.01		4.04 ± 1.71	
P4	1.87 ± 1.27		6.06 ± 2.08		4.18 ± 1.47	
Centro-parietal cluster	1.72 ± 1.17		5.99 ± 1.96		4.26 ± 1.55	

**Figure 2:**
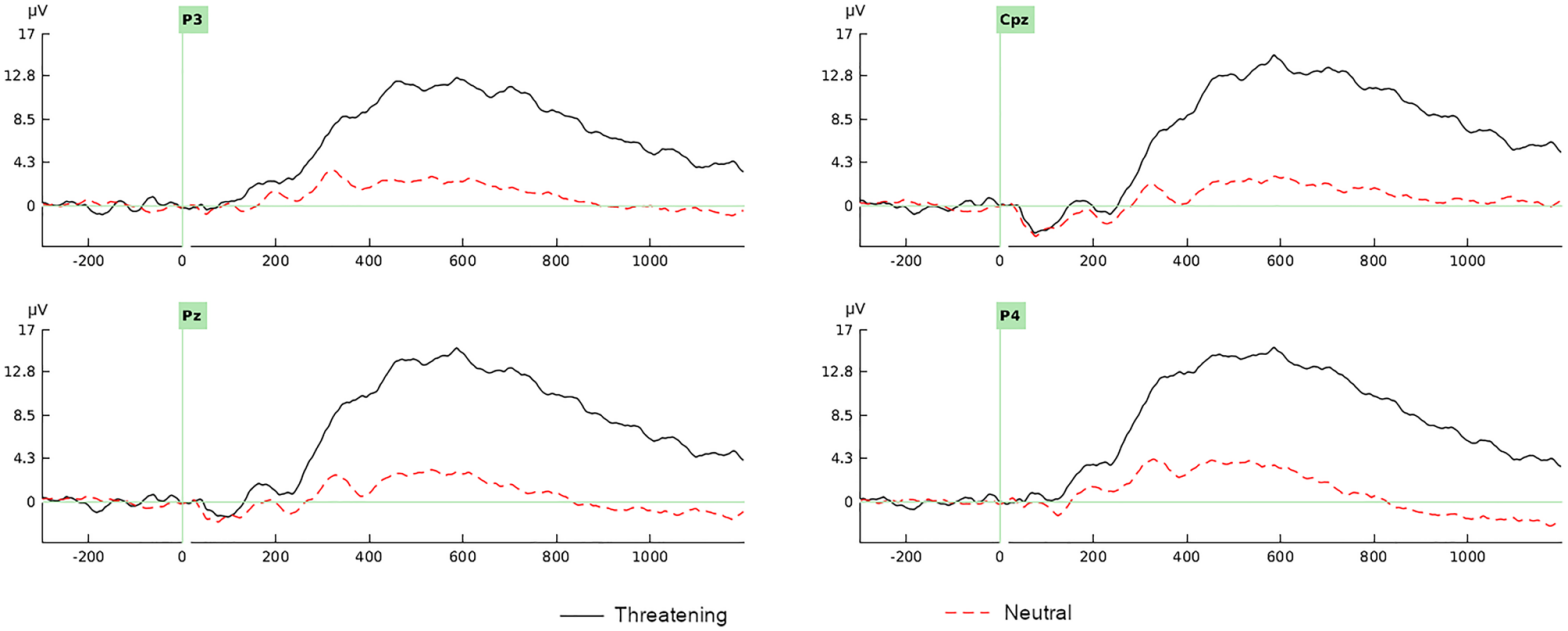
Grand average waveforms of LPP in the threatening (solid line) and neutral (broken line) conditions, in Cpz, Cz, P3 and P4 channels. Threatening stimuli were presented a time 0. LPP peak was defined as the most positive deflection with latency between 500 and 1000 ms post-stimulus interval. Positive is up.

**Figure 3:**
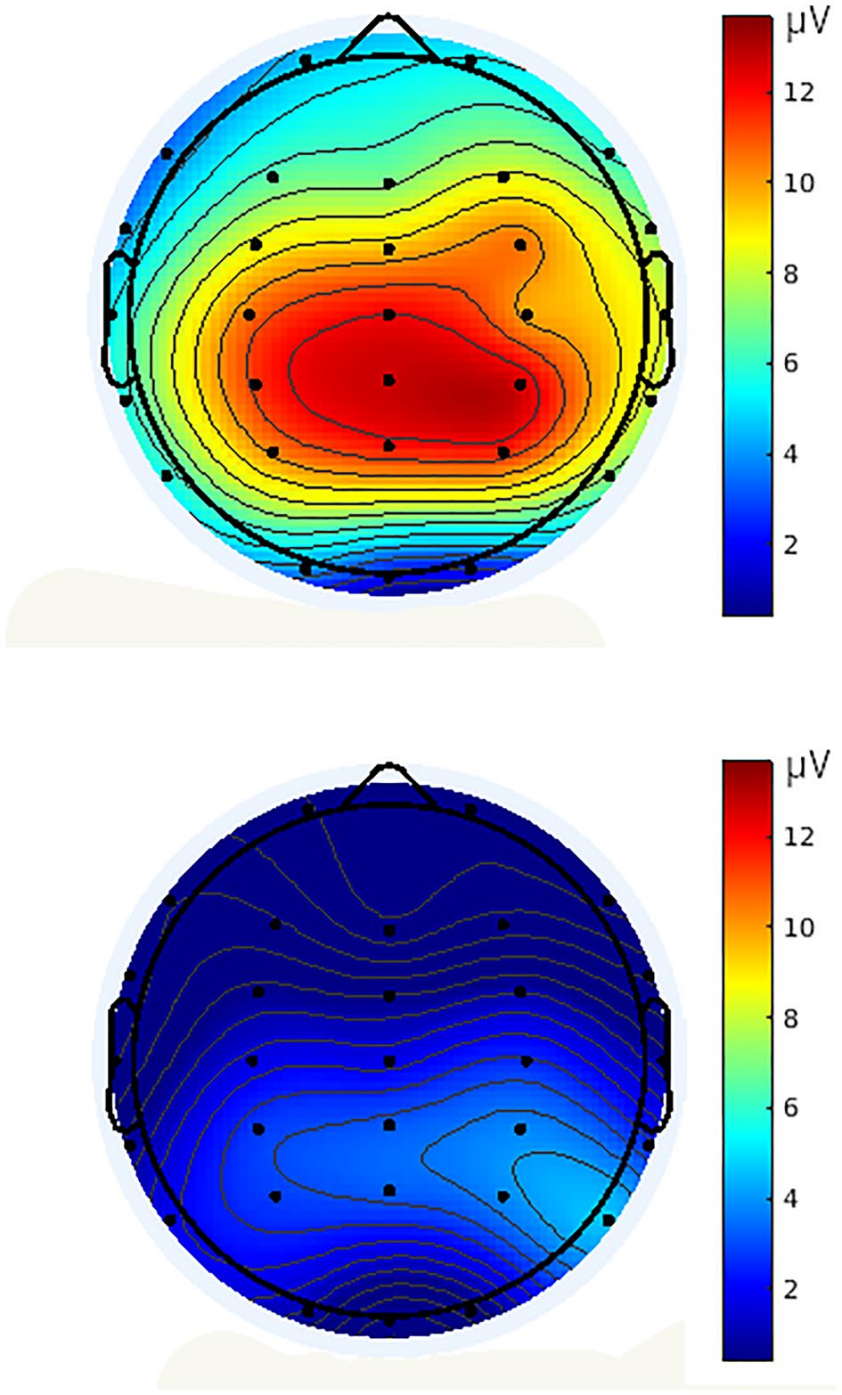
Topographic maps showing the scalp distributions of the LPP in the threatening (upper portion) and neutral (lower portion) conditions. The color bars show the voltage values of the component.

The two-way repeated measures ANOVA [2 (condition) × 4 (electrocortical position)] showed a significant increase in the threatening condition compared to the neutral condition *F*(1,51) = 394.897, *p* < 0.001, *η*^2^_*p*_ = 0.886. The main effect of electrocortical position with Huynh–Feldt correction was not significant *F*(2.600, 132.616) = 1.389, *p* = 0.251. The interaction effect was significant *F*(3,153) = 2.873, *p* = 0.038, *η*^2^_*p*_ = 0.053. (Table 1, Fig. 2). The exposure to the threatening stimuli produced a large amplitude in centro-parietal areas, but not between the electrocortical position, in comparison with the neutral condition (Fig. 3).

**Figure 4:**
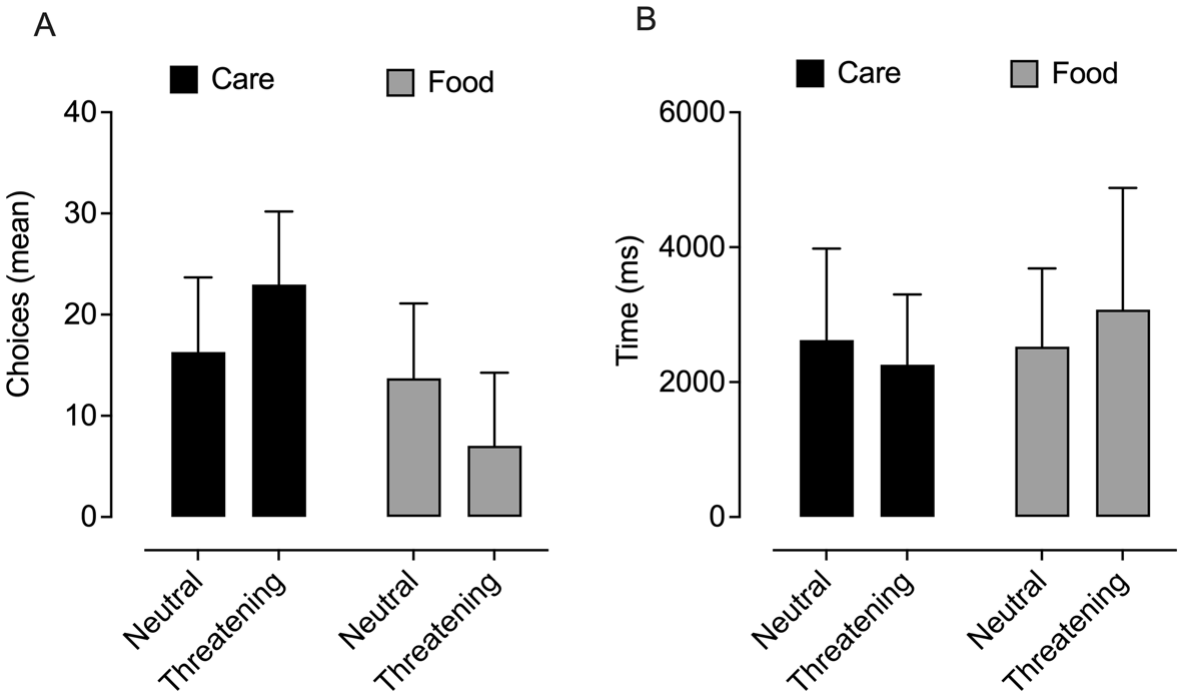
Differences in the choice of care or food (**A**) and respective RT (**B**) between threatening and neutral conditions. Data are expressed as mean ± SD.

The results of the repeated measures 2 × 2 ANOVA revealed the main effects of choice and condition (the choice of care vs. food are mutually exclusive) *F*(1,51) = 28.189, *p* < 0.001, *η*^2^_*p*_ = 0.356. The interaction effect was significant *F*(1,51) = 41.474, *p* < 0.001, *η*^2^_*p*_ = 0.448 (Fig. 4A). The simple effects shows that the differences between the two options of choice (care vs. food) were not significant in the neutral condition *F*(1,51) = 1.582, *p* = 0.214, ns. Whereas in the threatening condition a significant difference emerged *F*(1, 51) = 63.055, *p* < 0.001, *η*^2^_*p*_ = 0.553, with the percentage of care choice being about three times as high as food. With regard to the differences in the choice of care between the two conditions (neutral and threatening), the analysis showed that in the neutral condition participants chose the picture of care in about half of the cases (*M* = 16.29), while in the threatening condition the percentage increased by over 40% (*M* = 22.96). Averages of choices between the two conditions express significant differences *F*(1,51) = 41.474, *p* < 0.001, *η*^2^_*p*_ = 0.448. The choice of food mirrored the one of care.

The second 2 × 2 repeated analysis of variance yielded a main effect for the choice RT *F*(1,39) = 6.388, *p* = 0.016, *η*^2^_*p*_ = 0.141. The main effect of the condition was not significant *F*(1,39) = 2.530, *p* = 0.120, *ns*. The interaction effect was significant *F*(1,39) = 12.357, *p* = 0.001, *η*^2^_*p*_ = 0.241 (Fig. 4B). The simple effects analysis of RT show that participants choosing care in the threatening condition took significantly less time, compared with the food choice *F*(1,39) = 12.264, *p* < 0.001, *η*^2^_*p*_ = 0.239, while in the neutral condition the differences between care and food were not significant *F*(1,39) = 2.843, *p* = 0.100. It emerged that when participants chose food, the RT significantly increases in threatening with respect to neutral condition *F*(1,39) = 11.172, *p* = 0.002, *η*^2^_*p*_ = 0.223, while when they choose care the RT decrease in the threatening condition, but our result only reached near significance *F*(1,39) = 3.399, *p* = 0.073. It is to be noted that the degrees of freedom in the analysis of RT are lower, because 12 participants never chose food in the threatening condition.

The results of multivariate hierarchical regression that analyzed effects on choice of care are reported in Table 2.

**Table 2:** Predictors of care choice differences.

Variable	β	t	*R*	R^2^	*ΔR*^2^	*F* Change	*p*
Step 1			0.341	0.116	0.116	6.579	0.013
Care RT differences	− 0.341	− 2.565*					
Step 2			0.341	0.116	0.000	0.001	0.981
Care RT differences	− 0.341	− 2.512*					
Anxiety	0.003	0.024					
Step 3			0.444	0.197	0.081	4.854	0.032
Care RT differences	− 0.421	− 3.101**					
Anxiety	− 0.150	− 1.009					
Avoidance	0.339	2.203*					
Step 4			0.489	0.239	0.042	2.582	0.115
Care RT differences	− 0.491	− 3.495***					
Anxiety	− 0.126	− 0.858					
Avoidance	0.354	2.339*					
Centro-parietal cluster	0.216	1.607					

*p < 0.05, **p < 0.01, *** p < 0.001.

Step one indicates that when participants increased their choice of care in the threatening condition, as compared to the neutral condition, they decreased their RT. The inclusion of the attachment anxiety dimension (step two) provided no contribution to the choice model. Conversely, attachment avoidance contributed positively to the choice of care (step three). The results showed that high avoidance scores were associated with greater increases in care choice in the threatening condition. In step four, the inclusion of the centro-parietal cluster values did not produce a significant increase in the explained variance of the model. In fact, when all four independent variables were included in the regression model, neither attachment anxiety nor centro-parietal cluster were significant predictors of care choice differences. The model including the centro-parietal cluster explained 23.9% of the variance (R^2^ = 0.239) which was statistically significant, *F*(4,47) = 3.695, *p* = 0.011.

Results of the second multivariate hierarchical regression that analyzed the effects on care RT are reported in Table 3. Step one mirrored and confirmed the result of the first analysis on the association between RT and choice. In the step two we found that attachment anxiety, did not play a major role in the RT. Conversely, in step three we found that the inclusion of attachment avoidance contributed significantly to the time needed for the care choice decision. Participants with high avoidance tended to increase RT when choosing care in the threatening condition in comparison with the neutral condition. In step four, the effect of the centro-parietal cluster on RT in care choice, as measured by the difference between the two conditions, was positive and significant. The model with the four independent variables altogether, accounted for 35% of the variance (R^2^ = 0.350) in care RT,* F*(4,47) = 6.321,* p* < 0.001.

**Table 3:** Predictors of care decision time.

Variable	β	T	*R*	*R*^2^	Δ*R*^2^	F Change	*p*
Step 1			0.341	0.116	0.116	6.579	0.013
Care choice differences	− 0.341	− 2.565*					
Step 2			0.368	0.135	0.019	1.084	0.303
Care choice differences	− 0.334	− 2.512*					
Anxiety	0.138	1.041					
Step 3			0.495	0.245	0.109	6.936	0.011
Care choice differences	− 0.396	− 3.101**					
Anxiety	− 0.052	− 0.361					
Avoidance	0.385	2.634*					
Step 4			0.591	0.350	0.105	7.605	0.008
Care choice differences	− 0.420	− 3.495***					
Anxiety	− 0.021	− 0.153					
Avoidance	0.382	2.788**					
Centro-parietal cluster	0.327	2.758**					

*p < 0.05, **p < 0.01, ***p < 0.001.

The results of multivariate hierarchical regression that analyzed the effects of food on choice were not statistically significant. None of the independent variables provided a significant contribution to food choice. In addition, the results of multivariate hierarchical regression that analyzed effects on RT choice of food were not statistically significant.

## Discussion

The present study was designed to determine, firstly: the differences in LPP magnitude, care/food choices, and RT, between the threatening and the neutral condition; secondly, whether differences in the care choices and their RT were associated with specific attachment dimensions and differences of LPP magnitude in centro-parietal cluster.

In agreement with previous studies we found that the exposure to the threatening condition produced a larger LPP magnitude in centro-parietal cluster, in comparison with the neutral condition^43^. This result allowed testing the association between LPP magnitudes with the behavioral responses (choice and RT), which, in line with our hypothesis, confirms one of the core concepts of the attachment theory posits, i.e. that in a situation perceived as threatening, the motivational drive to seek care is activated^44^. While in the neutral condition care and food pictures were chosen almost equally, in the threatening condition the increased choice of care would perform an emotion regulation function. Lower RT values in the threatening condition, compared with the food choice, appear to express greater decisiveness when choosing care^6,45^.

In the present study, increased RT values were found in the threatening condition, which suggests that food, despite its regulatory function^31^ required a longer processing time before taking the decision of choosing it. Relevant research has shown that in general RT increases with the cognitive load of the task^46^, which could be the case of our threatening condition. Aside from the increase in RT, food choice showed no associations with attachment dimensions and LPP magnitude, and appeared to play a secondary role in comparison with care choice. The threatening condition in this study can be considered a stressful condition, and while some studies have associated food intake with acute stress^47^, other studies have associated it with chronic stress^48^. It is therefore likely that food, in addition to having a secondary regulatory role, could exert a stronger function in chronic stress than in acute stress, as in our experiment.

We also found that in the threatening condition the increased RT in care choice was associated to high LPP magnitudes. While in general the threatening condition promotes decreased RT in choosing care, this appears to be a prerogative of participants with low LPP magnitudes in the centro-parietal cluster. Greater amplitude in the same area in the threatening than neutral condition corresponded to increased RT in the choice of care (but not of food). These findings can be explained by an interference of the ongoing emotional process^49,50^ and could be interpreted in terms of aversion derived from threatening stimuli, following the neuro-anatomical model of attachment proposed by Long et al.^24^. Our hypothesis of the association between increased LLP and higher choice of care and increased RT in the threatening condition was partially confirmed. Indeed, increases in LLP in the centro-parietal cluster are associated with an increased, but not significant, choice of care.

While our data clearly showed that threatening stimuli increase LPP response and increment the choice of care, with respect with the neutral stimuli, we also studied the relationship between care choice, RT and attachment dimensions. The attachment theory has among its main cores the general tendency to seek care in threatening and dangerous situations. This aspect, regardless of the attachment style, is indeed clearly confirmed by our results. Unexpectedly, attachment anxiety did not show any association with increased choice of care and decreased RT in the threatening condition. In fact, anxiously attached individuals preferentially tend to heighten the effect of attachment-related stimuli due to their hypersensitivity, with anxious searching for care and support^51^. However, previous research did not find a clear association between attachment anxiety and proximity behavior^52^, showing that anxiously attached individuals (who worry that others will not be available in times of need^51^), tend to avoid exposure to threatening information^53^. According to these observations, our results may suggest that anxiously attached individuals will be initially vigilant and then will try to elude the threatening stimulus, in agreement with the vigilance-avoidance theory^54^.

Furthermore, various studies have also shown that avoidant attachment is characterized by a peculiar difficulty in seeking care in situations of need^6^, in particular because of the defenses built against the rejections received by their caregivers during the early stages of development^55^. On the other hand, previous researches have shown that when avoidant individuals are confident in the availability of their significant other, they are able to seek help and lower their defenses^32,56^. We found that high attachment avoidance is also associated with increased choice of care in the threatening condition. Indeed, the availability of care pictures may lead avoidant subjects to choose the care picture as a form of regulation, at the cost, however, of a significant increase in RT. The increase in care choice RT in avoidant subjects is in contrast to a general decrease in participants' RT in the threatening condition. This phenomenon has often been explained as greater attentional^57^ and cognitive engagement in the elaboration (e.g. avoidance defenses) of the choice process^15^, but also with an increase in distraction time due to an increased focus on the less emotional content of the threatening picture^58^. These avoidant defenses, (see dual process^9^), would operate as a cognitive load that increases RT. In other words, low avoidance and lower increased LPP amplitude produce a higher speed of decision.

### Limits and future directions

The first limit of the study we can identify is related to the nature and age of the population under analysis; in fact our sample was composed only of university students, although from different courses. Another limit may be related to the choice of comfort food as the only alternative to care. Although food is well recognized as a strong emotion regulator, other images of emotion regulation stimuli need to be used in future studies. Predictors have been selected based on theoretical assumptions. However other than the care RT criterion, the other criteria were found to be poorly correlated with the predictors. Still, the results in terms of more global models beyond individual correlations, are found to be significant.

We did not measure EEG data during the choice process, and this could also be a relevant aspect to be investigated in future studies. Further, functional connections studies should be useful in order to disentangle the role of the different brain areas during threatening emotional stimuli evaluation and emotion regulation choice process.

## Conclusions

Previous studies have demonstrated that threatening stimuli activate the centro-parietal area of the brain in comparison to neutral stimuli. Our results show that while the choice of care in the threatening condition is generally associated with a decrease in RT, an increase in the centro-parietal amplitude is associated with an increase in care RT. The involvement of the centro-parietal area is shown to be associated with specific regulatory responses, in particular relational ones. It is therefore confirmed that the propensity is to use care (and not food) as a form of emotional regulation, despite the strong role of food in emotional regulation. When considering the dimensions of attachment, anxiety does not show associations neither with the choice of care/food or with decision-making times. On the contrary, avoidance is associated with an increase in the choice of care in the threatening condition and to an increase in RT, in contrast with the decrease in RT shown by the total sample. This data shows how in certain situations the defenses of avoidant subjects can be by-passed, and the increased RT may reflect as a more articulated elaboration of the choice process.

## Data Availability

The datasets generated during and/or analysed during the current study are available from the corresponding author A.U. on reasonable request.
