# Author Correction: ERP and attachment dimensions as predictors of seeking care or food comfort in stressful situations

**DOI:** 10.1038/s41598-023-32430-w

**Published:** 2023-03-31

**Authors:** Arcangelo Uccula, Beniamina Mercante, Caterina Pozzati, Franca Deriu, Paolo Enrico

**Affiliations:** 1 Department of History, Human Sciences and Education, University of Sassari, Via Zanfarino 62, 07100 Sassari, Italy; 2 Department of Biomedical Sciences, University of Sassari, Viale San Pietro 43/B, 07100 Sassari, Italy; 3 Unit of Endocrinology, Nutritional and Metabolic Disorders, AOU Sassari, Sassari, Italy

Correction to: *Scientific Reports* 10.1038/s41598-023-29493-0, published online 23 February 2023

The original version of this Article contained an error in the name of authors Arcangelo Uccula, Beniamina Mercante, Caterina Pozzati, Franca Deriu and Paolo Enrico, which were incorrectly given as Uccula Arcangelo, Mercante Beniamina, Pozzati Caterina, Deriu Franca and Enrico Paolo.

The original Article has been corrected.

